# Mechanism and treatment of intracerebral hemorrhage focus on mitochondrial permeability transition pore

**DOI:** 10.3389/fnmol.2024.1423132

**Published:** 2024-07-31

**Authors:** Jing Cong, Jing-Yi Li, Wei Zou

**Affiliations:** ^1^The First School of Clinical Medicine, Heilongjiang University of Chinese Medicine, Harbin, China; ^2^The Second School of Clinical Medicine, Heilongjiang University of Chinese Medicine, Harbin, China; ^3^Molecular Biology Laboratory of Clinical Integrated of Traditional Chinese and Western Medicine of Heilong Jiang Province, The First Affiliated Hospital of Heilongjiang University of Chinese Medicine, Harbin, China

**Keywords:** cerebral hemorrhage, mitochondria, mitochondrial permeability transition pore, treatment, mechanism

## Abstract

Intracerebral hemorrhage (ICH) is the second most common subtype of stroke, characterized by high mortality and a poor prognosis. Despite various treatment methods, there has been limited improvement in the prognosis of ICH over the past decades. Therefore, it is imperative to identify a feasible treatment strategy for ICH. Mitochondria are organelles present in most eukaryotic cells and serve as the primary sites for aerobic respiration and energy production. Under unfavorable cellular conditions, mitochondria can induce changes in permeability through the opening of the mitochondrial permeability transition pore (mPTP), ultimately leading to mitochondrial dysfunction and contributing to various diseases. Recent studies have demonstrated that mPTP plays a role in the pathological processes associated with several neurodegenerative diseases including Parkinson’s disease, Alzheimer’s disease, Huntington’s disease, ischemic stroke and ischemia-reperfusion injury, among others. However, there is limited research on mPTP involvement specifically in ICH. Therefore, this study comprehensively examines the pathological processes associated with mPTP in terms of oxidative stress, apoptosis, necrosis, autophagy, ferroptosis, and other related mechanisms to elucidate the potential mechanism underlying mPTP involvement in ICH. This research aims to provide novel insights for the treatment of secondary injury after ICH.

## 1 Introduction

Spontaneous intracerebral hemorrhage (ICH), characterized by non-traumatic parenchymal bleeding (de Oliveira Manoel et al., 2016), represents the second most prevalent stroke subtype (Qureshi et al., 2009) and exhibits a substantial mortality rate (Feigin et al., 2009), with 30–55% mortality within 30 days (Balami and Buchan, 2012). Survivors often face an unfavorable prognosis, frequently accompanied by diverse neurological deficits. Despite numerous studies conducted over the past decades to enhance secondary injury management following ICH, significant improvements in patient outcomes have unfortunately not been achieved (Roger et al., 2011). Consequently, it is imperative to explore novel avenues for treating secondary injury subsequent to ICH.

Mitochondria, a double-layered membrane-coated organelle found in the majority of eukaryotic cells, serve as the primary site for aerobic respiration and act as the energy production hub within cells. Mitochondrial dynamics play a crucial role in body development, particularly in the brain (Flippo and Strack, 2017) and heart (Dorn et al., 2015), while also influencing stem cell self-renewal and differentiation (Seo et al., 2018). Under pathological conditions, mitochondria can induce the opening of mPTP, leading to alterations in mitochondrial permeability that result in mitochondrial dysfunction. This dysfunction often initiates a cascade of cellular death processes and contributes to various diseases. Recent studies have demonstrated that mPTP is implicated in the pathological progression of several neurological disorders such as Parkinson’s disease (Rasheed et al., 2017), Alzheimer’s disease (Jia and Du, 2021), Huntington’s disease (Quintanilla et al., 2017), ischemic stroke (Yangxin et al., 2020) and ischemia-reperfusion injury (Norbert et al., 2016), among others. However, there is limited research on mPTP regarding ICH. Therefore, this article aims to review both physiological and pathological processes associated with mPTP, elucidate its relationship with ICH, explore potential mechanisms by which mPTP contributes to secondary injury following ICH, and propose new avenues for research into treating secondary injury after ICH.

## 2 Composition of the mPTP

**FIGURE 1:**
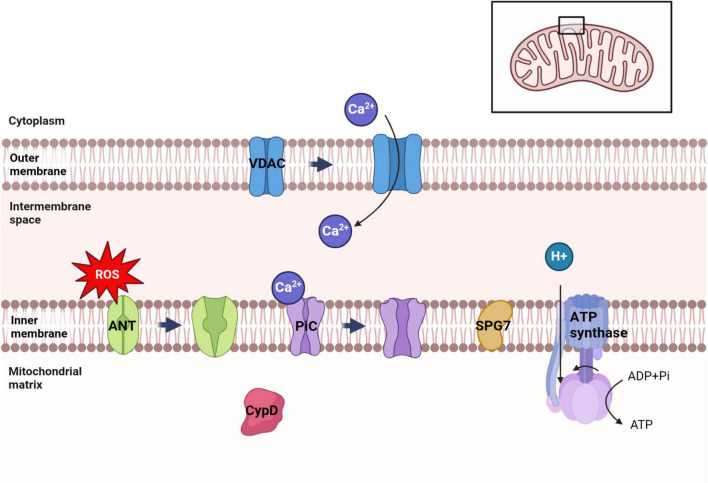
Distribution of several major candidate proteins of mPTP. VDAC was distributed in the outer membrane of mitochondria. ANT, PiC, SPG7 and ATP synthase were distributed in the inner membrane of mitochondria. CypD is distributed in the mitochondrial matrix. Among them, VDAC can regulate ATP transport and Ca2+ uptake in mitochondria. Ca2+ can trigger the conformational changes of ANT and PiC, leading to the opening of mPTP. ATP synthase uses the electrochemical membrane gradient of protons to drive the rotation of its own subunits and catalyze ATP synthesis to provide energy to the cell.

**FIGURE 2:**
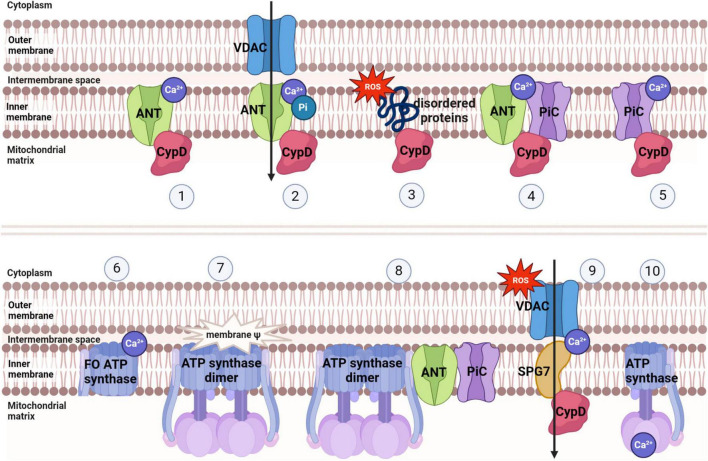
Several models of mPTP and factors inducing mPTP opening.

Mitochondria, being crucial organelles in the body, participate in a diverse range of cellular processes and are also exposed to various biochemical stimuli, such as oxidative stress. Under these adverse conditions, the permeability of the mitochondrial inner membrane undergoes changes known as mitochondria permeability transition (MPT). Initially believed to be caused by alterations in the phospholipid bilayer of mitochondria, further research has revealed that this permeability transition is triggered by the opening of protein channels on both the inner and outer mitochondrial membranes (Crompton et al., 1987). These protein-based channels are referred to as mPTP. The composition of mPTP has been a subject of controversy since its proposal in 1987. Currently, several models for mPTP have been proposed by scholars (as shown in Table 1). Among them, major candidates for mPTP composition include proteins like cyclophilin D (CypD), adenine nucleotide translocase (ANT), voltage-dependent anion channel (VDAC), mitochondrial phosphate carrier (PiC), ATP synthase, and Paraplegin (SPG7) (as showed in Figures 1, 2).

**TABLE 1:** Several current models of mPTP.

Proteins	Condition	Cell culture methods	References
CypD-ANT	The conformational changes of ANT triggered by Ca^2+^ in the matrix	Incubate the isolated rat liver and heart mitochondria in 150 mM-KSCN or sucrose medium	Halestrap and Davidson, 1990
VDAC-CypD- ANT	Ca^2+^ and Pi, binding allow flicker into an open pore form.	The cDNA encoding cyclophilin-D was cloned from a rat liver library and expressed in Escherichia coli XL1 cells.	Crompton et al., 1998
Misfolding and clustering of native membrane proteins-Chaperone-like proteins-CypD	Oxidative damage and other perturbations (e.g., Ca^2+^)	Incubate isolated rat liver mitochondria in sucrose medium	He and Lemasters, 2002
PiC-ANT-CypD	Triggered by calcium, promoted by CypD, regulated by the conformational state of ANT molecules, and caused by conformational changes in PiC	Incubate isolated rat liver and heart mitochondria, as well as bovine heart mitochondria, in sucrose medium	Leung et al., 2008
PiC-CypD	Ca^2+^	Incubate isolated rat liver and heart mitochondria, as well as bovine heart mitochondria, in sucrose medium	Leung and Halestrap, 2008
FO ATP synthase	The c subunit of the FO ATP synthase plays a critical role in the CsA-dependent opening of the PTPC induced by cytosolic Ca^2+^ overload.	Glycolysis and respiratory cell model of human cervical carcinoma HeLa cells	Bonora et al., 2013
Dimers of mitochondrial ATP synthase	Membrane potential and substrate pH are key regulatory factors for ATP synthase	Glycolysis and respiratory cell models of mitochondria in bovine or mouse heart and human osteosarcoma ahqb17 cells	Giorgio et al., 2013
Dimers of mitochondrial ATP synthase-ANT- PiC	Stress conditions	Hypothesis	Gutiérrez-Aguilar and Baines, 2015
SPG7-CypD- VDAC	ROS, Ca^2+^	An experimental system using HEK293T and HeLa cells was developed, in which expression of mitochondrial proteins was silenced individually by short hairpin RNA and evaluated for Ca^2+^- and ROS-induced PTP opening.	Shanmughapriya et al., 2015
FO ATP synthase forms a Ca^2+^-dependent high- conductance channel	Ca^2+^	FO ATP synthase was purified from bovine heart mitochondria by a combination of sucrose density gradient centrifugation and ion-exchange chromatography employing the mild.	Mnatsakanyan et al., 2019; Urbani et al., 2019

Cyclophilin is a peptidyl-prolyl cis-trans isomerase that is widely distributed across various species in nature. In the human body, there are 16 cyclophilins, with CypA, CypB, and CypD being the most extensively studied (Fischer et al., 1989; Wang and Heitman, 2005). Unlike other cyclophilins, CypD exhibits unique localization within the mitochondrial matrix. Initially considered as a specific cell membrane binding protein, CypD’s activity can be inhibited by the immunosuppressant cyclosporin A (CSA) (Crompton et al., 1988). It has been discovered that CSA can block the opening of mPTP by inhibiting CypD activity. This inhibition prevents the binding of CypD to other mitochondrial membrane proteins and consequently affects mitochondrial pore formation. Recent studies have reported that several mitochondrial membrane proteins including VDAC (Crompton et al., 1998), ANT (Halestrap and Davidson, 1990), PiC (Leung and Halestrap, 2008), and ATP synthase (Bonora et al., 2013) can interact with CypD to form mPTP. Therefore, it is evident that CypD plays a crucial role in regulating mPT. Research has demonstrated that loss of CypD expression can inhibit mPTP opening, reduce cell necrosis, protect cardiomyocytes, and improve cardiac function (Lam et al., 2015). Down-regulation of CypD expression also mitigates neuronal damage caused by amyloid-β and oxidative stress while reducing aging-related conditions (Lin and Beal, 2006) as well as Alzheimer’s disease occurrence (Du et al., 2008).

ANT is a member of the mitochondrial carrier family protein, which constitutes 10% of the total mitochondrial protein content (Brand et al., 2005). It is synthesized in the cytoplasm and subsequently inserted into the inner mitochondrial membrane (Ryan et al., 1999). ANT primarily functions as a transporter for metabolites and cofactors on the mitochondrial inner membrane, but its main role is to facilitate ADP/ATP exchange on this membrane (Palmieri, 2004), thereby generating charge difference on it (Duszyński et al., 1981). Although ANT can affect mPTP opening, its exact role in this process remains controversial. Early studies suggested that Ca^2++^ could trigger conformational changes in ANT, promote binding between ANT and CypD, and then cause mPTP opening (Halestrap and Davidson, 1990). It has also been proposed that under certain conditions, VDAC-CypD-ANT forms a multiprotein complex that leads to mPTP opening (Crompton et al., 1998). However, subsequent studies found that Ca^2+^-induced mPTP opening could still occur even if activity of ANT isoforms was inhibited. Therefore, some scholars believe that rather than participating in composition of mPTP itself, ANT plays a regulatory role in its opening process instead (Leung and Halestrap, 2008).

VDAC, also known as mitochondrial porin, belongs to the eukaryotic mitochondrial porin family. It is situated in the outer membrane of mitochondria and regulates molecular and ion exchange between the cytoplasm and mitochondria, thereby controlling mitochondrial metabolites (Shoshan-Barmatz et al., 2010). VDAC plays a role in cellular energy metabolism by regulating the transport of ATP or ADP across the inner and outer membranes of mitochondria (Maldonado and Lemasters, 2014). Additionally, it modulates mitochondrial calcium uptake (Rosencrans et al., 2021) and is involved in biological processes such as apoptosis (Mazure, 2017), autophagy (Lemasters, 2007), and ferroptosis (Zhao et al., 2020). While VDAC’s status as a key component of mPTP remains controversial (Shanmughapriya et al., 2015), its association with mPTP opening is undeniable. Research has demonstrated that the interaction between α-synuclein (α-SYN) and VDAC1 promotes mPTP opening, which can lead to Cytc release and mitochondrial swelling (Shen et al., 2014); this may be one of the pathological mechanisms underlying certain neurodegenerative diseases.

One of the primary functions of mitochondria is to provide energy (ATP) through oxidative phosphorylation, which necessitates the importation of ADP and inorganic phosphate (Pi) across the inner mitochondrial membrane (Ernster and Schatz, 1981). The transport of ADP and Pi requires the involvement of Mitochondrial carrier proteins. Similar to ANT, PiC also belongs to the mitochondrial carrier family protein (Gao et al., 2020), predominantly found in cardiac and skeletal muscle tissues (Seifert et al., 2015). Its main responsibility lies in replenishing consumed Pi within mitochondria either through OH-exchange or proton binding mechanisms (Stappen and Krämer, 1994). It has been reported that PiC plays a crucial role in mPTP formation, with changes in its conformation induced by Ca^2+^ leading to mPTP opening (Seifert et al., 2016). Studies have demonstrated that increased levels of PiC caused by a high Pi environment result in elevated mitochondrial superoxide production and vascular smooth muscle cell death. However, inhibition of PiC reduces mitochondrial Pi uptake while inhibiting mitochondrial oxidative stress and ERK1/2 activation (Thi Nguyen et al., 2023).

The mitochondrial F1FO-ATP synthetase, also known as ATP synthase, is a large multisubunit protein complex (greater than 500 kDa) that consists of an FO domain, a peripheral stalk, and a soluble F1 domain comprising a catalytic head and a central stalk (Pinke et al., 2020; Spikes et al., 2020). It belongs to the rotating ATPase family and is widely distributed in the energy transduction membranes of bacteria, chloroplasts, and mitochondria (Zharova et al., 2023). This enzyme facilitates the synthesis of ATP from ADP and inorganic phosphate through the final step of oxidative phosphorylation (or photophosphorylation), which is a fundamental pathway for energy production in animal, plant, and microbial cells (Trchounian and Trchounian, 2019). While its primary function is ATP synthesis, under certain pathological and physiological conditions it can also hydrolyze ATP to provide energy to the inner mitochondrial membrane. Additionally, there have been suggestions that ATP synthase contributes to cell death by participating in the opening of mPTP. Under normal circumstances, ATP synthase utilizes the electrochemical transmembrane gradient of protons to drive FO rotation and initiate ATP synthesis at the F1 catalytic center. However, upon mPTP opening leading to depolarization of the mitochondrial inner membrane and loss of proton transmembrane electrochemical gradient necessary for FO rotation initiation occurs resulting in limited ATP synthesis ultimately leading to cell death (Mnatsakanyan et al., 2019).

As a mitochondrial protease, SPG7 is responsible for ribosome assembly and the elimination of misfolded proteins within mitochondria (Koppen et al., 2007). Mutations in SPG7 result in the misformation of the protease complex, leading to defective protein quality control, impaired mitochondrial function, and compromised neuronal signaling. Consequently, this gives rise to a wide range of diseases including spastic paraplegia (Casari et al., 1998), ataxia (Pfeffer et al., 2015), amyotrophic lateral sclerosis (Osmanovic et al., 2020), among others. It has been suggested that SPG7 acts as a core component of mPTP and interacts with VDAC1 and CyPD. Knockout studies have shown that the absence of SPG7 impairs ion efflux from mitochondria to cytoplasm and disrupts calcium retention capacity (Shanmughapriya et al., 2015). However, Bernardi and Forte (2015) propose that SPG7 indirectly regulates rather than serves as a core component for mPTP opening. Recently, genetic research reported that SPG7 neither forms pores in mitochondrial permeability transition nor regulates mPTP opening (Klutho et al., 2020). Therefore, further investigation is required to determine the role of SPG7 in mPTP.

## 3 Conditions for opening the mPTP

The researchers have made a precise and bold prediction of the mPTP model, postulating that MPTS are induced by two distinct types of pores, each facilitating the passage of two currents: Firstly, a low conductance state with an amplitude ranging from 0.3 to 0.7 nS, allowing for the permeation of ions (such as protons, Ca^2+,^ and K^+^) and small metabolites (e.g., glutathione) across the inner mitochondrial membrane under physiological conditions. Secondly, a high conductance state with an amplitude of approximately 1.5 nS, enabling the transit of large solutes (like sucrose) (Neginskaya et al., 2019). However, this latter scenario exerts detrimental effects on mitochondrial structure and function leading to irreversible permanent permeability opening and ultimately regulated cell death (RCD).

Under normal physiological conditions, the low conductance state triggers opening due to the accumulation of Ca^2+^. Upon redistribution of ions between the mitochondria and the cytoplasm, H^+^ ions enter the mitochondria from the cytoplasm, causing rapid closure of mPTP by reducing the pH of the mitochondrial matrix. This spontaneous transition between on and off states of mPTP is referred to as “flicker” (Boyman et al., 2019). The flicker of mPTP is a physiological phenomenon that provides an additional mechanism for effluxing Ca^2+^ from the mitochondrial matrix, mitigates persistent overload of Ca^2+^ in the matrix, regulates Ca^2+^ reserves within mitochondria, and further modulates Ca^2+^-related metabolism (Bernardi and von Stockum, 2012). Moreover, the flicker can facilitate transportation of endogenous ROS and other molecules generated in mitochondria into the cytoplasm; however, this may serve as a self-protective mechanism for mitochondria (Zorov et al., 2000).

Unlike flicker, prolonged opening of mPTP can have detrimental effects on mitochondrial physiology and energy metabolism. When the mPTP is opened to a high conductance, the redistribution of ions and small molecules leads to a reverse redistribution of H_2_O. The entry of water into the mitochondrial matrix increases osmotic pressure, resulting in partial expansion of mitochondrial cristae and an increase in matrix volume. This causes mitochondrial swelling accompanied by mechanical rupture of the mitochondrial outer membrane (Bonora et al., 2022). Opening of high-conductance mPTP typically occurs under pathological conditions and often leads to difficult-to-repair damage in mitochondria. Pathological stimuli such as Ca^2+^ overload, oxidative stress, increased phosphate concentration, and decreased adenine nucleotide availability can promote the high permeability of the mitochondrial inner membrane to macromolecular solutes (Halestrap, 2009; Morciano et al., 2015; Boyenle et al., 2022). The substantial non-selective influx of these high molecular solutes and sudden loss of metabolites within mitochondria disrupts mitochondrial homeostasis, ultimately leading to programmed cell death (Izzo et al., 2016).

The opening of mPTP induced by Ca^2+^ overload has been widely acknowledged by scholars. Studies have demonstrated that Ca^2+^-treated mitochondria can generate reactive oxygen species (ROS) and hydrogen peroxide (H_2_O_2_), which damage the membrane components in mitochondria and induce mPT (Kowaltowski et al., 2001). Mc Stay et al. discovered that Cys160 and Cys257 sites of ANT were oxidatively modified in Ca^2+^-treated mitochondria, significantly increasing the affinity of cyclophilin D to ANT and inducing mPTP generation (McStay et al., 2002). Mitochondrial pH also serves as a precise regulator of mPT production. An acidic environment inhibits the opening of mPTP since H^+^ ions replace Ca^2+^ and bind to the trigger site of mPTP (Halestrap, 1991). Oxidative stress and Pi concentration are two other known inducers for mPTP opening. Mitochondria are an important source of ROS (Giorgi et al., 2018), and pro-oxidants facilitate Ca^2+^-induced mPTP opening. Stimulation with Pi can induce mPT and promote ROS generation, leading to enhanced oxidative stress response, further facilitating Pi-induced mPT. This situation reduces the threshold for mPTP opening, making mitochondrial cells prone to spontaneous occurrence of mPT (Kanno et al., 2004). Proapoptotic members Bax, Bak, and Bad from the Bcl-2 family have been reported to regulate mPTP opening. Bax/Bak depletion in mitochondria renders cells resistant to mPTP opening and necrosis; however, recombination of these cells or mitochondria with wild-type Bax can restore susceptibility possibly due to bax-driven cell fusion lowering the threshold form PTP opening (Whelan et al., 2012). Roy et al. proposed that stress factors such as ceramide and huperzine can dephosphorylate Bad by activating protein phosphatase 2A (PP2A), allowing dephosphorylated Bad to firmly bind to the mitochondrial membrane and interact with Bcl. This process shifts VDAC and makes mPTP sensitive to Ca^2+,^ promoting its opening (Roy et al., 2009).

## 4 Cellular consequences of mPTP opening

Under pathological conditions, the continuous and extensive opening of mPTP disrupts the internal mitochondrial environment and alters mitochondrial osmotic pressure, leading to a cascade of irreversible damage to both mitochondria and the organism as a whole. The persistent mPTP opening triggers the release of ROS, resulting in oxidative stress and subsequent mitochondrial dysfunction (Liu E. et al., 2020; Wacquier et al., 2020). ROS accumulation damages nuclear DNA, activates pro-apoptotic signaling pathways, and induces cellular senescence (Zorov et al., 2014; Liu et al., 2022). Changes in osmotic pressure can cause mitochondria to swell and rupture, ultimately leading to their lysis and demise while regulating cell necrosis (Mnatsakanyan et al., 2017). Furthermore, mPTP opening is also implicated in processes such as mitophagy (Baechler et al., 2019), ferroptosis (Basit et al., 2017), neutrophil extracellular traps (NETs) (Yang et al., 2021), pyroptosis (Zhang et al., 2022b), and inflammatory response (Bonora et al., 2022).

### 4.1 Oxidative stress

Under physiological conditions, the balance between production and scavenging of ROS is tightly regulated. Depending on the context, controlled oxidative stress can elicit various cellular responses, ranging from activating signaling pathways involved in cytoprotection to initiating coordinated activation of mitochondrial fission and autophagy for efficient clearance of abnormal mitochondria and cells, while preventing damage propagation to neighboring organelles and cells (Bernardi and Di Lisa, 2015; Briston et al., 2017). Conversely, uncontrolled oxidative or reductive stress can result in severe cellular damage and unnecessary cell death, leading to organ failure at both local and systemic levels (Szeto, 2008; Bernardi et al., 2015). In normal physiological conditions, ROS release accounts for approximately 2% of total mitochondrial oxygen consumption (Peuhkurinen et al., 1983). Both low and high ratios of ROS release can have detrimental effects: insufficient ROS release fails to fulfill proper cellular functions; excessive ROS release lacks a regulatory effect. As pivotal organelles governing cellular metabolism and redox homeostasis, mitochondria possess precise mechanisms to regulate ROS levels according to cellular demands (Cadenas, 2018).

However, under certain pathological conditions, the structure and function of mitochondria can become impaired. Mitochondria not only serve as the primary producers of ROS, but also actively contribute to their harmful amplification. Research has demonstrated that electron leakage from mitochondria accounts for approximately 90% of ROS generation (Zheng et al., 2022). The mPTP may play a pivotal role in this cascade of events. mPTP activation promotes ROS production, which in turn leads to further opening of the mPTP; this positive feedback mechanism ultimately results in excessive ROS accumulation and subsequent detrimental effects on mitochondrial function (Gareev et al., 2023).

Changes in cellular redox balance are intricately linked to the respiratory activity of mitochondria (Korshunov et al., 1997). Numerous post-translational modifications have been documented in oxidative phosphorylation complexes, aiding in the regulation of ROS production (Covian and Balaban, 2012). For instance, tyrosine phosphorylation of cytochrome oxidase and cytochrome c serves to inhibit respiration and safeguard mitochondria against hyperpolarization and subsequent elevation of ROS levels (Hüttemann et al., 2012). However, ROS can disrupt the association between cytochrome c and mitochondrial inner membrane cardiolipin, impairing its ability to shuttle electrons from complex III to complex IV. Consequently, electron accumulation occurs within respiratory complexes I and III, further fueling mitochondrial ROS generation. Once mitochondrial ROS reaches a certain threshold level, an influx of Ca^2+^ ensues depleting the robust redox buffering capacity within mitochondria, leading to uncontrolled escalation of mitochondrial ROS (Bernardi et al., 2015). Simultaneously, diffused ROS can affect neighboring mitochondria that have not undergone mPT thereby reducing the threshold for mPTP opening there and initiating a detrimental cycle ultimately resulting in extensive cellular ROS production (Zorov et al., 2014) and deleterious oxidative stress (Bonora et al., 2022).

### 4.2 Apoptosis

When mitochondrial permeability is impaired, the oxidative stress response becomes interconnected with subsequent damage to both mitochondria and cells, ultimately leading to apoptosis. Recent research has demonstrated that an elevation in reactive ROS production occurs prior to DNA fragmentation-induced apoptosis (Golstein and Kroemer, 2007). Various pathological factors causing cellular hypoxia result in reduced oxygen levels, thereby diminishing mitochondrial ATP production and triggering an increase in intracellular Ca^2+^ concentrations. Concurrently, hypoxia disrupts the functionality of the mitochondrial electron transport chain, leading to heightened ROS generation. Elevated levels of Ca^2+^ and ROS contribute to the opening of the mPTP, further exacerbating Ca^2+^ and ROS accumulation within mitochondria while promoting protein and lipid oxidation within these organelles. The resultant calcium overload and oxidative stress induce mitochondrial dysfunction, subsequently initiating apoptosis (Yao et al., 2022).

Mitochondria serve as the regulatory centers of apoptosis, and multiple lines of evidence suggest that mitochondria-mediated opening of mPTP plays a crucial role in initiating apoptosis (Chakraborti et al., 1999; Lin and Beal, 2006). Upon the opening of mPTP, cytochrome c and its associated proteins are released from mitochondria. However, it is widely acknowledged that the release of cytochrome c from mitochondria represents a pivotal step in apoptosis. Subsequently, cytochrome c released into the cytoplasm binds to apoptosis-associated factor 1 (Apaf-1) in the presence of ATP, leading to polymer formation and subsequent binding with caspase-9 to form apoptotic bodies. Activation of caspase-9 then triggers a cascade effect by activating other caspases such as caspase-3. Consequently, this process promotes cellular apoptosis (Zoratti and Szabò, 1995).

The production of ROS by mitochondria has been demonstrated to oxidize crucial thiol groups of the mPTP-related component ANT, thereby triggering the release of cytochrome c and initiating a cascade of events leading to apoptosis (Kanno et al., 2004). Furthermore, previous studies have suggested that polythiols, but not monothiols, selectively inhibit the release of cytochrome c (Nishikimi et al., 2001). Subsequent investigations have revealed that adjacent dithiol and/or two proximal thiol residues in ANT and related proteins within the mitochondrial membrane may underlie Ca^2+^-induced cytochrome c release. This study proposes that Ca^2+^ enhances the cross-linking between two thiol groups, such as Cys160 and Cys257, in ANT, resulting in the liberation of cytochrome c from mPTP (Kanno et al., 2004).

The VDAC protein is located in the outer mitochondrial membrane and was previously believed to be a crucial component of mPTP, although this assertion remains controversial (Shanmughapriya et al., 2015). The activation or deactivation state of VDAC plays a significant role in regulating apoptosis within mitochondria. The N-terminal α-helix domain of VDAC1 is indispensable for stabilizing its original open state; however, it has been reported that the N-terminal region can also form a pore large enough to facilitate the release of cytochrome c from mitochondria, subsequently triggering apoptosis (Abu-Hamad et al., 2009). Interestingly, even when in the closed state, VDAC can induce apoptosis by promoting calcium ion influx into mitochondria and subsequent opening of mPTP (Tan and Colombini, 2007). Moreover, previous studies have demonstrated that overexpression of VDAC1 can initiate apoptosis (Zaid et al., 2005; Weisthal et al., 2014). Under normal circumstances, VDAC exists in various oligomerization states (Shoshan-Barmatz et al., 2013), with overexpression leading to the formation of mPTP and subsequent release of cytochrome c, ultimately resulting in cell apoptosis (Keinan et al., 2010; Khan et al., 2021).

### 4.3 Necrosis

The process of cell necrosis is distinct from apoptosis, which is a form of programmed cell death. While apoptosis is regulated, cell necrosis is considered to be unregulated; however, some scholars argue that a significant portion of necrosis occurs through highly regulated mechanisms as well. During cell necrosis, the intracellular ATP level decreases to a point where the cell can no longer maintain its physiological activities. This leads to vacuolar appearance in the cytoplasm, damage to the cell membrane, and overflow of cellular contents including broken organelles and chromatin fragments. Consequently, this triggers an inflammatory response in surrounding tissues (Golstein and Kroemer, 2007; Kung et al., 2011).

Although apoptosis and necrosis are mediated by different pathways, there are instances where their production pathways overlap and apoptotic processes can further contribute to the occurrence of necrosis (Golstein and Kroemer, 2007; Chipuk et al., 2010). In mitochondria, caspase activation plays a key role in apoptosis due to the release of cytochrome c and other apoptotic factors. However, primary necrosis does not require involvement of cytochrome c. Nevertheless, if clearance of apoptotic bodies is delayed or absent following apoptosis, secondary necrosis ensues (Kung et al., 2011).

The opening of mPTP triggers mitochondrial membrane depolarization, resulting in the cessation of mitochondrial ATP synthesis and an influx of water into the solute-rich mitochondrial matrix. Consequently, osmotic pressure builds up between the matrix and the protein-dense extramitochondrial environment, leading to matrix swelling and crista folding and stretching, ultimately culminating in mitochondrial swelling, rupture, and initiation of cell death (Lam et al., 2013; Shibata et al., 2019). Furthermore, this swelling-induced dilution hampers enzyme activity and metabolite concentration within mitochondria, impairing tricarboxylic acid (TCA) cycle function as well as respiratory complex activities. As a consequence, ATP synthase ceases ATP synthesis at this stage, causing a rapid blockade of ATP-dependent reactions. Under these circumstances, mitochondria are unable to create conditions necessary for mPTP deactivation; thus, rendering it irreversible while further accelerating cell death onset.

Additionally, extensive opening of the mPTP results in a cellular energy crisis, leading to an uncontrolled escalation in glycolysis rate. This heightened glycolytic activity causes pyruvate accumulation, resulting in acidification of both the cytoplasmic and mitochondrial matrix. Consequently, the plasma membrane ATPase actively extrudes Na^+^, Ca^2+^, and H^+^ ions from the cytosol, while the sarcoplasmic/endoplasmic reticulum Ca^2+^-ATPase (SERCA) pumps Ca^2+^ into the endoplasmic reticulum. Subsequently, there is a disappearance of Na^+^ and Ca^2+^ gradients along with a deceleration in H^+^ pumping, exacerbating cytoplasmic acidification. The profound loss of ion gradients ultimately culminates in plasma membrane collapse and necrotic cell death (Galluzzi et al., 2018).

### 4.4 Autophagy

Autophagy is a catabolic process that selectively or non-selectively degrades dysfunctional or unnecessary cellular components in eukaryotic cells, thereby maintaining cellular homeostasis and facilitating stress response under normal conditions (Batoko et al., 2017). Nonselective autophagy serves as a protective mechanism by providing cells with biomolecular fuel to generate energy during periods of starvation (Rabinowitz and White, 2010). However, autophagy also plays a role in regulating cell death when cells are exposed to injury-induced stress or signaling stimulation (Galluzzi et al., 2015; Zhao et al., 2017).

Mitophagy refers to the process through which mitochondria or parts of mitochondria are targeted for degradation within lysosomes (Montava-Garriga and Ganley, 2020; Nguyen and Lazarou, 2021). The occurrence of autophagy leads to internal cristae fragmentation, protein degradation, and even retention of only the outer membrane structure (Minibayeva et al., 2012). Mitophagy occurs conservatively and specifically, serving as an important self-regulatory pathway for maintaining cellular stability.

According to the current study, specific proteins on the surface of mitochondria appear to function as markers for autophagic recruitment in mitochondria, while mPTP plays a crucial role as a component of the mitochondrial membrane. The opening of mPTP can trigger alterations in autophagic activity (Rodriguez-Enriquez et al., 2006). Electron transport chain (ETC), an integral part of mitochondria, generates low levels of ROS to facilitate cell signaling (Maldonado and Lemasters, 2014; Huang et al., 2016). Through redox reactions that generate ATP in the oxidative phosphorylation system, they establish an electrochemical gradient. Under hypoxic conditions, cellular oxidative metabolism and energy are disrupted, leading to mitochondrial dysfunction and inadequate ATP supply along with excessive ROS production (Zelinová et al., 2019). The generation of ROS induces autophagy while also promoting mPTP opening and continuous ETC impairment (Gomes and Scorrano, 2013), further exacerbating autophagy. Numerous studies have demonstrated that mitochondria undergoing permeability transitions are often targeted by autophagy. Extensive intracellular mPT triggers excessive mitophagy, resulting in programmed cell death (Van Aken et al., 2016). In cases where mammalian hepatocytes experience nutrient starvation, MPT occurrence leads to an accelerated depolarization rate of the mitochondrial membrane and subsequently promotes mitophagy (Reumann et al., 2010).

When mitochondria are damaged, the damaged mitochondria are often transported to lysosomes for degradation, a process frequently observed in the activation of the PINK1-parkin pathway (Palikaras et al., 2018). This mechanism is believed to mediate mPTP-induced mitophagy following deletion of the mitochondrial fission protein DRP1 (Song M. et al., 2015). Fused mitochondria facilitate more efficient diffusion of Ca^2+^ within them, which stimulates mPTP opening (Baumgartner et al., 2009), an event disrupted by Drp1-mediated mitochondrial fission. Inhibition of Drp1-mediated fission results in morphologically elongated mitochondria and increased mPTP opening (Szabadkai et al., 2004). The opening of mPTP can cause depolarization of the mitochondrial membrane, leading to accumulation of PINK1 on the mitochondrial surface and subsequent initiation of mitophagy, ultimately resulting in autophagosome uptake and release (Basit et al., 2017). However, in the absence of DRP1, inhibition of mPTP opening reduces mitochondrial lysosomal phagocytosis while increasing mitochondrial content (Baumgartner et al., 2009).

The involvement of mPTP in autophagy can also occur via the apoptotic pathway. Mitochondrial outer membrane permeability is regulated by BCL2 family proteins, which govern the intrinsic apoptotic pathway. It has been demonstrated that mPTP facilitates the translocation of pro-apoptotic protein Bax to the outer mitochondrial membrane. The anti-apoptotic protein BCL2 forms a heterodimer with BAX to regulate cellular apoptosis. Furthermore, BCL2 interacts with BECN1/Beclin 1 to modulate autophagy. However, phosphorylation of BCL2 enhances its binding affinity for Bax and dissociates it from BECN1, thereby inducing autophagy (Wei et al., 2008). These findings underscore the pivotal role of both BCL2 and mPTP in regulating the delicate balance between apoptosis and autophagy through their regulation of Bax (Liu et al., 2018).

Additionally, mitophagy also impacts mPT, and the accumulation of dysfunctional mitochondria induced by autophagy results in a reduction in mitochondrial membrane potential and an elevation in ROS production. This scenario decreases the threshold for mPTP opening, rendering mitophagy-deficient cells susceptible to spontaneous mPT (Baechler et al., 2019; Sun et al., 2019), thus perpetuating a vicious cycle. In animals, the mitochondrial protease PARL inhibits autophagy through Omi activation, also known as HtrA2, thereby preserving cristae structure and preventing mPTP opening and cytochrome c release (Chao et al., 2008).

### 4.5 Ferroptosis

Different from apoptosis, necrosis, and autophagy, ferroptosis is a regulated mode of cell death that relies on iron ions. Its primary characteristic is the accumulation of lipid peroxides (Dixon et al., 2012; Xie et al., 2016). The process of lipid peroxidation can be initiated by enzymatic or non-enzymatic reactions catalyzed by lipoxygenase. Once generated, lipid peroxide reacts with Fe^2+^, oxygen, or surrounding unsaturated fatty acids, leading to continuous generation of free radicals and their diffusion into the surrounding area, ultimately triggering cell death (Tang et al., 2021).

Although unsaturated fatty acids in the cell membrane undergo continuous oxidation, lipid peroxidation and ferroptosis can be inhibited by antioxidant mechanisms under physiological conditions. Glutathione peroxidase 4 (GPX4) plays a crucial role in this process by catalyzing the reaction between glutathione GSH and lipid hydroperoxides, thereby reducing lipid peroxidation to lipid alcohols and preventing the accumulation of lipid peroxides, thus protecting cells (Friedmann Angeli et al., 2014; Conrad et al., 2016). However, the functional activity of GPX4 is regulated by system XC- in the plasma membrane. System xc- encodes heterodimeric amino acid transport through SLC7A11 (xCT) and SLC3A2 (4F2hc), which facilitates cystine-glutamate exchange (Ying and Padanilam, 2016). It transports extracellular cystine into cells where it is converted to cysteine−a necessary substrate for maintaining GPX4 activity. Impaired cystine transport can result in decreased intracellular GSH levels and increased ROS production (Montero et al., 2013), consequently leading to the accumulation of lipid hydroperoxides (Friedmann Angeli et al., 2014).

Currently, an increasing body of research provides crucial evidence implicating mitochondrial damage in the process of iron-induced cell death. Jelinek et al. discovered that exposure to RSL3 induced iron death in neuronal HT 22 cells and mouse embryonic fibroblasts, resulting in a decrease in GPX4 concentration, increased lipid peroxidation and mitochondrial fragmentation, decreased mitochondrial membrane potential and respiration. However, the use of iron death inhibitors reversed these effects. Furthermore, the application of ROS scavengers effectively preserved the integrity of mitochondrial morphology and partially inhibited the occurrence of iron-induced cell death (Jelinek et al., 2018).

An important intersection between mitochondrial dysfunction and iron-induced cell death is the activation of mPTP. It has been observed that Erastin-induced iron-mediated cell death in SH-SY5Y cells is accompanied by a decline in mitochondrial membrane potential, intracellular ATP content, and an accumulation of oxidative stress markers. However, treatment with cyclosporin A can prevent Erastin-induced mitochondrial changes and subsequent cell death by binding to CypD. This suggests that mPTP activation plays a crucial role in iron-induced cell death. Interestingly, it should be noted that ROS production gradually increases over time, while cyclosporin A significantly inhibits this process only during later stages. There is reason to believe that ROS accumulation during these later stages may be triggered by mPTP activation, whereas initial ROS production induced by erastin could potentially result from GSH depletion (Ganguly et al., 2024).

Studies have revealed that mitophagy can trigger uncontrolled degradation of mitochondria, resulting in the release of stored iron and subsequent iron overload-induced ferroptosis (Du et al., 2020). Throughout this process, mitochondria undergo a series of alterations including volume atrophy, outer membrane rupture, crista shrinkage, inner membrane compression, and electron transparent nucleus formation (Xie et al., 2016). Ultimately, these changes culminate in membrane disruption, depolarization of mitochondrial membrane potential, and mPTP opening. However, the activation of mPTP leads to ROS generation which exacerbates mitochondrial energy dysfunction and organelle swelling/rupture while inducing characteristic ferroptotic cell death, thus establishing a vicious cycle (Kwong and Molkentin, 2015; Basit et al., 2017). Additionally demonstrated is that oxidative stress triggers p53 translocation to mitochondria in Bax/Bak double knockout mice’s isolated mitochondria. This translocation induces CypD-p53 complex formation which promotes mPTP opening and consequent cell death (Vaseva et al., 2012). Simultaneously activated p53 renders cells more susceptible to ferroptosis by inhibiting SLC7A11 transcription resulting in decreased cystine uptake along with reduced GSH levels leading to an additional increase in ROS production ultimately driving ferroptotic cell demise (Zhang et al., 2018).

As a crucial component of mPTP, VDAC1 plays a pivotal role in the occurrence of ferroptosis, contributing to tissue damage and organ dysfunction associated with various diseases (Niu et al., 2022). Studies have demonstrated that Tanshinone IIA (TSN), an active compound extracted from Danshen, can effectively mitigate myocardial cell injury by suppressing ferroptosis during anoxia/reoxygenation (A/R). Pre-treatment with TSN leads to upregulation of genes involved in ferroptosis inhibition, reduction in total iron content, lipid peroxide levels, and superoxide dismutase expression while inhibiting ferroptotic activation. However, overexpression of VDAC1 can induce mPTP opening and counteract the protective effect exerted by TSN on cells. This suggests that TSN prevents A/R-induced ferroptosis by downregulating VDAC1 expression to prevent mPTP opening (Hu et al., 2023).

### 4.6 Others

The regulation of inflammation and the opening of mPTP are interconnected (Bonora et al., 2022). Induction of mPTP triggers the activation of inflammatory response through succinate release and the release of damage-associated molecular patterns (DAMPs) associated with mitochondrial damage, thereby leading to tissue damage following cerebral ischemia (Tian H. Y. et al., 2023). The opening of mPTP induces ferroptosis, which in turn leads to secretion of HMGB1, a DAMP that activates pattern recognition receptor (PRR)/NF-κB pathway, resulting in an inflammatory response in peripheral macrophages (Chen et al., 2021). It has been observed that under stress conditions, mitochondrial DNA (mtDNA) is released into the cytoplasm via mPTP, activating cyclic GMP-AMP synthetase (cGAS)-stimulator of interferon genes (STING) pathway and exacerbating downstream inflammatory responses. Genetic deletion or pharmacological inhibition of STING as well as inhibition of mtDNA release reduce inflammatory responses; however, transfection with mtDNA supports cytoplasmic mtDNA-induced inflammatory responses by activating cGAS-STING pathway (Ouyang et al., 2023). Deletion of CypD, a major component of mPTP, attenuates proinflammatory cytokine expression in mouse islets (Liu et al., 2019) and mitigates inflammatory responses induced by exposure to bacterial products such as lipopolysaccharide in macrophages or liver cells (Veres et al., 2021).

The aging process is associated with significant alterations in mitochondrial function. These changes in mitochondrial function are believed to be linked to an increased production of ROS, which contribute to cellular death, senescence, tissue degeneration, and impaired tissue repair over time. The opening of the mPTP may play a crucial role in these processes, as elevated ROS levels activate mPTP opening, further exacerbating ROS production (Zorov et al., 2014). Injury and inflammation are also thought to enhance mPTP opening, and chronic low-grade inflammation is a characteristic feature of aging. Nicotinamide adenine dinucleotide (NAD+) inhibits the frequency and duration of mPTP opening; however, NAD+ levels decline with age, thereby promoting mPTP opening and increasing ROS release. Accumulation of ROS can damage nuclear DNA, trigger pro-apoptotic signaling pathways, and drive cellular senescence (Schaar et al., 2015; Fang et al., 2016). Mitophagy regulates senescent cells by removing fragmented mitochondria with activated mPTP; nevertheless, autophagy is inhibited in senescent cells leading to increased activation of mPTP. However, during aging the heightened activity of mPTP makes autophagy a detrimental process that contributes to cellular senescence and demise (Rottenberg and Hoek, 2021).

NETs are unique structures formed by neutrophils outside the cell, composed of DNA, histones, and granule contents. NETs represent an inflammatory form of cell death in neutrophils. Research has demonstrated that SIRT1 can induce the activation of mPTP channels and release mitochondrial DNA, leading to the formation of mitochondria-dependent NETs which subsequently initiate a cascade of pathological reactions (Yang et al., 2021).

NLRP3 inflammasome-mediated pyroptosis is an inflammatory programmed cell death process that promotes chronic inflammation and tissue degeneration. Human nucleus pulposus cells exposed to oxidative stress conditions exhibit characteristic morphological and functional changes associated with mPTP opening and cytoplasmic release of mtDNA. Inhibiting mPTP opening specifically or preventing mtDNA release into the cytoplasm effectively reduces NLRP3 inflammasome-mediated pyroptosis in nucleus pulposus cells as well as inflammation within the microenvironment in vitro, ultimately mitigating degenerative progression in a rat intervertebral disc acupuncture model (Zhang et al., 2022a).

## 5 mPTP participating in the pathological process of ICH

**FIGURE 3:**
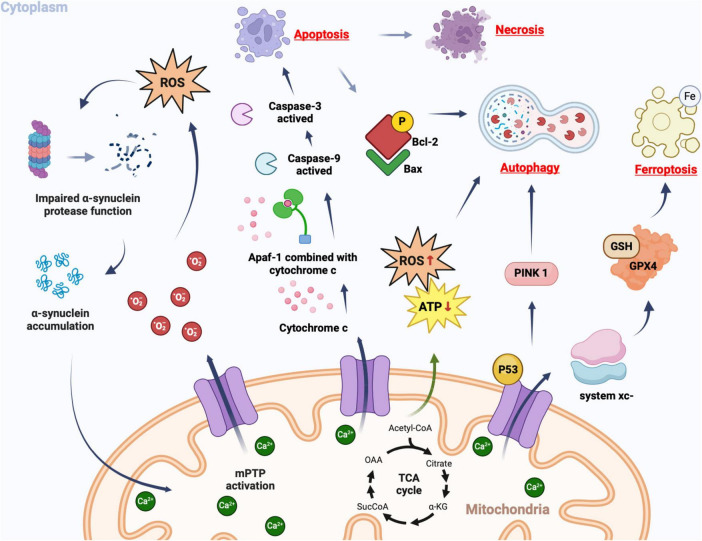
Cellular consequences of mPTP opening. In pathological conditions, mPT promotes the production of ROS, which in turn leads to further opening of the mPTP. Simultaneously, ROS can impair proteomic clearance of α-synuclein, resulting in its accumulation and interaction with mitochondria, thereby further activating mPTP opening. This creates a vicious cycle that triggers oxidative stress reactions. Upon mPTP opening, cytochrome c is released from mitochondria and binds to apoptosis-related factor 1 (Apaf-1), facilitating caspase-9 binding and subsequent activation of caspase-3, ultimately triggering apoptosis. If apoptotic bodies are not promptly cleared, secondary cell necrosis occurs immediately after apoptosis. The opening of mPTP can cause ATP supply insufficiency and excessive ROS production, inducing autophagy. Mitochondrial damage-induced accumulation of PINK1 further promotes autophagy. Phosphorylated Bcl-2 following mPTP opening can induce autophagy by binding to Bax protein. The opening of mPTP results in decreased GPX4 concentration and affects the regulation system xc-, as well as GSH expression catalyzed by it; this reduction impairs cystine transport leading to lipid hydroperoxide accumulation and subsequently iron death.

The pathological process following ICH can be categorized into two stages: primary brain injury and secondary brain injury. The former refers to the mechanical damage caused by the space-occupying effect of the hematoma, while the latter encompasses a series of pathological processes involving excitatory amino acid toxicity, reactive oxygen species, vascular inflammatory cell-mediated tissue oxidative stress, apoptosis, necrosis, autophagy, and so forth. Ultimately leading to severe neurological deficits (Wang Z. et al., 2018; Chen et al., 2020; Zheng et al., 2023). Currently, there is substantial evidence linking the opening of mPTP with the pathological process after ICH (as depicted in Figure 3).

### 5.1 ICH and oxidative stress

Due to its high lipid and iron content and relatively low levels of antioxidants, the brain is susceptible to damage from oxidative stress (OS) (Xu et al., 2018). Numerous studies have demonstrated the association between OS and the pathophysiology of various brain disorders (Salim, 2017), particularly ICH. OS can be triggered by ICH and plays a crucial role in secondary brain injury following such an event (Wang S. et al., 2018). Increasing evidence suggests that ROS levels increase while antioxidant enzyme activity decreases in the brain after ICH (Xie et al., 2017). Experimental models of ICH have shown elevated levels of ROS and malondialdehyde (a marker of oxidative stress) in rats (Liu X. C. et al., 2020). During ICH, an excessive amount of ROS disrupts the delicate balance between the antioxidant system and ROS, resulting in cellular damage including lipid peroxidation, DNA damage, protein oxidation, apoptosis induction, blood-brain barrier (BBB) breakdown and overall brain impairment (Han et al., 2008; Katsu et al., 2010).

Multiple mechanisms can mediate the generation of ROS following ICH, and glutamate is among them. Glutamate serves as a contributing factor to nervous system injury by inducing excitotoxicity in neurons. Following ICH, a substantial release of glutamate occurs from the blood, activating NMDA receptors and resulting in an influx of Ca^2+^. This excessive increase in cytoplasmic Ca^2+^ subsequently leads to mitochondrial Ca^2+^ overload. Additionally, thrombin production after ICH activates Src kinase, leading to phosphorylation of NMDA receptors and further enhancement of their function (Sharp et al., 2008). However, mitochondrial Ca^2+^ overload causes a decrease in transmembrane potential and opening of mPTP, thereby damaging mitochondria and the respiratory chain, ultimately leading to increased ROS release (Mracsko and Veltkamp, 2014). Furthermore, this destructive damage has the ability to propagate from one mitochondrion to another (Qu et al., 2016), resulting in continuous expansion of the oxidative stress response.

### 5.2 ICH and apoptosis

The occurrence of apoptosis following ICH is widely observed in the cerebral cortex, subcortical and hippocampal neurons. Additionally, traces of apoptosis can even be detected within the cerebrovascular system alongside brain parenchyma. Studies have demonstrated that vascular endothelial cell apoptosis directly exacerbates vasospasm severity post-intracerebral hemorrhage and further intensifies secondary injury (Zhou et al., 2004). ICH, as an external stress event, can trigger apoptosis through various mechanisms (Hasegawa et al., 2011). The mitochondria-related apoptotic pathway plays a crucial role in secondary injury after ICH (Fujii et al., 2013). Mitochondria are highly dynamic organelles that continuously undergo fusion and division to adapt to changes in energy demand and supply while maintaining cellular function (Zhou et al., 2021). Under normal circumstances, the dynamic balance between mitochondrial fusion and division is a pivotal event regulating mitochondrial morphology for ensuring normal mitochondrial function (Giacomello et al., 2020). However, following ICH, there is abnormal regulation of mitochondrial dynamics with an imbalance between fusion and fission processes leading to a gradual shift towards fission (Wu et al., 2020b). Mounting evidence suggests that excessive mitochondrial division serves as a key factor contributing to ICH development (Wu et al., 2020a; Zhang et al., 2021). Excessive fission disrupts the structural integrity of mitochondria. During this process, a series of morphological changes can be observed, including prominent mitochondrial clustering, spherical shrinkage, loss of integrity, mitochondrial swelling, darkening of the matrix, and irregular cristae formation (Cheng et al., 2021; Zhang et al., 2021). Consequently, mitochondrial oxidative phosphorylation is impaired while ROS production increases. This leads to decreased ATP levels and mitochondrial membrane potential ultimately resulting in mitochondrial dysfunction (Wu et al., 2020a), which also serves as an early indicator of apoptosis. The heightened mitochondrial fission prolongs mPTP opening (Chang et al., 2021), leading to sustained release of apoptotic factors (such as cytochrome C) from mitochondria. Subsequently, activation of the caspase cascade occurs followed by apoptosis (Azarashvili et al., 2016).

The protein p53 has been demonstrated to play a crucial role in coordinating the apoptotic pathway following ICH (Cahill and Zhang, 2009; Lauzier et al., 2023). Upon occurrence of ICH, p53 activates the mitochondrial apoptotic pathway via the Bcl-2 protein family, which subsequently modulates the release of cytochrome C from mPTP based on the prevailing signal (i.e., pro-apoptotic or anti-apoptotic dominance) (Philchenkov, 2004). Moreover, under oxidative stress induced by ROS, p53 accumulates within the mitochondrial matrix and interacts with CypD to trigger mPTP opening, thereby initiating a detrimental cycle of enhanced ROS release and apoptosis induction (Vaseva et al., 2012; Munro and Treberg, 2017).

### 5.3 ICH and necrosis

As previously discussed, apoptosis is a delayed form of cell death that occurs as a result of less severe injury and is associated with the activation of a “genetic program.” In contrast, necrosis is a rapid response to severe injury such as hypoxia and cell trauma. Despite their morphological differences, there is increasing evidence suggesting the existence of similar mechanisms for apoptosis and bad cell color, both of which are linked to the opening of mPTP. Following ICH, the opening of mPTP can directly lead to mitochondrial dysfunction and neuronal damage (Banasiak et al., 2000).

Under normal circumstances, mitochondria primarily play a crucial role in supplying cellular energy. However, when mitochondrial dysfunction occurs, it can lead to various consequences in neuronal damage, ranging from temporary metabolic inhibition to cell necrosis depending on the severity of mitochondrial impairment (Yu et al., 2019). Following ICH, the release of glutamate triggers neuronal necrosis, and this specific mechanism is associated with mitochondrial dysfunction (Nicotera and Lipton, 1999). A subset of cerebellar granule cells was observed to undergo necrotic death during and shortly after exposure to glutamate. These neurons experienced a collapse in mitochondrial membrane potential, swelling of the nucleus, and dispersion of intracellular debris into the culture medium. Glutamate stimulation resulted in decreased mitochondrial membrane potential and ATP levels in numerous neurons. In cases where there was irreversible dissipation of the mitochondrial membrane potential, rapid necrosis occurred (Nicotera and Lipton, 1999). Insufficient ATP production leading to reduced intracellular energy can further contribute to cytotoxic edema development and cell necrosis. Neurons have higher energy demands compared to other cells; therefore, inadequate energy supply causes more significant damage to neurons (Prentice et al., 2015). Additionally, mitochondria can induce ROS production. The accumulation of ROS disrupts the pro-oxidation-antioxidant balance within cells and contributes to oxidative stress occurrence—ROS being a key factor in neuronal cell necrosis (Prentice et al., 2015). Henceforth, maintaining the integrity of both mitochondrial morphology and function plays a vital role in protecting nerve cells following ICH; mPTP serves as a critical determinant influencing this process.

### 5.4 ICH and autophagy

Autophagy is a vital lysosomal degradation and recycling process in eukaryotic cells, responsible for maintaining cellular function, homeostasis, and the delicate balance between cell survival and death (Tian Z. et al., 2023). Studies have demonstrated the sensitivity of vascular smooth muscle cells (VSMCs) to alterations in autophagic regulation (Salabei and Hill, 2015). Autophagy is indispensable for VSMC survival, as knockdown of autophagy-related genes increases their susceptibility to cell death (Grootaert et al., 2018). Furthermore, autophagy plays a significant role in various cardiovascular and cerebrovascular diseases such as atherosclerosis (Shao et al., 2016) and ischemic stroke (Mo et al., 2020). Recent evidence has also implicated autophagy in secondary injury following ICH (Zhang and Liu, 2020). Miro1, a mitochondrial transport protein highly expressed after ICH, exhibits superior maintenance of mitochondrial membrane potential and promotes mitochondrial health. Consequently, researchers speculate that Miro1 may safeguard neurons by regulating intercellular mitochondrial transport to mitigate secondary injury post-cerebral hemorrhage (Li B. et al., 2021). Additionally, Miro1 serves as a substrate for the E3 ubiquitin ligase Parkin. The PINK1-Parkin pathway induces mitophagy by triggering mPTP opening (Song M. et al., 2015), which is crucial for eliminating damaged mitochondria subsequent to ICH occurrence (Kazlauskaite et al., 2014).

The microtubule-associated protein 1 light chain 3 (LC3) serves as a specific autophagy marker, appearing exclusively during the initiation of phagocytosis and exhibiting correlation with ROS expression (Tooze and Yoshimori, 2010). Studies have demonstrated that VDAC1, a crucial component of mPTP, co-localizes with LC3 following ICH. Positive staining for TUNEL and propidium iodide PI indicates that VDAC1 (mPTP) plays a pivotal role in inducing mitophagy, apoptosis, and necrosis. Inhibition of VDAC by VDAC 1 siRNA attenuates LC3 expression while upregulating ROS protein accumulation, histological manifestations of apoptosis and necrosis, as well as caspase-3 protein levels. Conversely, activation of autophagy through rapamycin significantly enhances LC3 expression while reducing ROS protein expression and abolishing histological manifestations of apoptosis and necrosis (Li et al., 2014). This finding offers a novel direction for future treatment strategies targeting secondary injury in ICH.

### 5.5 ICH and ferroptosis

As a form of iron-dependent programmed cell death, ferroptosis can be triggered by lipid peroxidation (Stockwell et al., 2017; Guo et al., 2020). However, both the iron and lipid pathways are implicated in the pathological process following ICH. Early studies have demonstrated that iron, a major component of hemoglobin, is highly neurotoxic and can be released during hematoma formation after ICH to initiate the Fenton reaction. The lipid pathway can induce the generation of various hydroxyl radicals, thereby promoting tissue oxidation, damaging cell membranes, DNA and proteins, ultimately leading to ferroptotic death of nerve cells (Djulbegovic and Uversky, 2019; He et al., 2020). Studies have revealed that ICH can result in diverse forms of neuronal cell death with ferroptosis accounting for over 80% of total neuronal deaths in ICH models; thus, highlighting its indispensable role in ICH pathology (Li Y. et al., 2021). During the acute phase of ICH, microglia and infiltrating macrophages phagocytose and decompose hemoglobin (Hu et al., 2016; Wan et al., 2019), resulting in substantial production of extracellular iron biliverdin and carbon monoxide (Zhou et al., 2020), which further facilitates intracerebral hematoma development (Chen et al., 2019). This process leads to mitochondrial damage causing excessive reactive oxygen species production (Lemasters, 2017) while disrupting glutamine metabolism leading to impaired antioxidant pathways and hydroxyl radical synthesis ultimately culminating in ferroptotic demise of nerve cells (Magtanong and Dixon, 2018; DeGregorio-Rocasolano et al., 2019).

Cheng et al. proposed a close association between mitochondria and ferroptosis following ICH. Their study revealed impaired mitochondrial function in both human and murine models of ICH, with specific inhibitors targeting mitochondria triggering and exacerbating neuronal ferroptosis, thereby worsening neurological deficits post-ICH (Cheng et al., 2023). Furthermore, this study identified the most prominent morphological characteristic of ferroptotic cells as alterations in mitochondrial morphology observed under electron microscopy (Dixon et al., 2012). Post-ferroptosis, there was a reduction in mitochondrial volume, increased concentration of mitochondrial membrane density, thinning or disappearance of mitochondrial cristae, and rupture of the outer mitochondrial membrane. These distinct changes make monitoring mitochondrial morphological alterations an effective method for assessing ferroptosis both in vitro and in vivo alongside measuring cellular levels of ROS and iron (Xie et al., 2016).

### 5.6 Neuronal axons and mPTP

A substantial body of evidence supports the essential role of mitochondrial functional integrity in neuronal survival following ICH (Diao et al., 2020). Mitochondrial dysfunction contributes to a cascade of secondary injuries after ICH, primarily due to their involvement in eliminating damaged mitochondria and repairing injured neurons (Zhou et al., 2017). Several studies have indicated that axonal injury after ICH is predominantly caused by mitochondrial dysfunction resulting from mPTP opening, which offers a novel avenue for investigating axonal degeneration post-ICH (Yang et al., 2022). Axonal injury mainly occurs in the extra-hematoma parenchyma following ICH and generally exhibits an increasing trend during the initial three days post-ICH (Wasserman and Schlichter, 2008). As the primary energy generators within neuronal cells, mitochondria play a crucial role in regulating nutrient transport and physiological functions of neuronal axons (Chang et al., 2006; Nikić et al., 2011). The survival of axonal and dendritic neuronal fibers is heavily reliant on mitochondrial transport, as mitochondria provide sufficient energy to distal synapses for normal ion channel, transporter, and synaptic transmission activities (Chang et al., 2006). Mitochondria are now considered fundamental components of axon elongation/regeneration and branching (Smith and Gallo, 2018). Mitochondrial damage not only reduces ATP release but also increases axonal degeneration (Smith and Gallo, 2018). The interaction between microtubules and actin filaments mediates axonal extension and guidance. Studies have found that microtubule replacement and mitochondrial dysfunction are the initial events of axonal injury after ICH. ATP deficiency caused by mitochondrial dysfunction and mPTP opening is a crucial initial link in axonal injury that directly leads to microtubule disassembly (Li et al., 2022).

## 6 Explore the treatment of ICH from the perspective of mPTP

The latest research has revealed that mitochondrial dysfunction and the subsequent opening of mPTP play a pivotal role in the occurrence of secondary injury following ICH. Consequently, targeting mPTP as a means to enhance EBI represents a promising therapeutic avenue.

mPTP is implicated in various cellular death processes associated with ICH, and targeting specific components of mPTP may serve as an effective approach to enhance EBI. VDAC functions as a channel-forming protein situated in the outer mitochondrial membrane, facilitating the passage of metabolites and iron across the outer membrane (Hodge and Colombini, 1997; Colombini, 2012). Partial inhibition of VDAC hampers mitochondrial metabolism and diminishes mitochondrial membrane potential (Maldonado and Lemasters, 2012; Yao et al., 2018). The ferroptosis activator erastin can induce VDAC opening, facilitate mitochondrial iron uptake, elevate mitochondrial membrane potential, leading to depolarization of the mitochondria and subsequent induction of neuronal death through mitochondrial dysfunction. However, targeted application of VDAC inhibitors can reverse the occurrence of EBI (Ismael et al., 2020). Therefore, stable expression of VADC can be employed as an effective target for EBI treatment. mPTP is implicated in various cellular death processes associated with ICH, and targeting specific components of mPTP may serve as an effective approach to enhance EBI. VDAC functions as a channel-forming protein situated in the outer mitochondrial membrane, facilitating the passage of metabolites and iron across the outer membrane (Hodge and Colombini, 1997; Colombini, 2012). Partial inhibition of VDAC hampers mitochondrial metabolism and diminishes mitochondrial membrane potential (Hodge and Colombini, 1997; Colombini, 2012). The ferroptosis activator erastin can induce VDAC opening, facilitate mitochondrial iron uptake, elevate mitochondrial membrane potential, leading to depolarization of the mitochondria and subsequent induction of neuronal death through mitochondrial dysfunction. However, targeted application of VDAC inhibitors can reverse the occurrence of EBI (Hodge and Colombini, 1997; Colombini, 2012). Therefore, stable expression of VADC can be employed as an effective target for EBI treatment. Additionally, targeted modulation of CypD can confer a significant protective effect on the organism. It has been observed that endogenous SO2 can sulfonate the Cys104 residue of CypD, thereby impeding the opening mechanism of mPTP and effectively inhibiting apoptosis (Boyang et al., 2022).

The neuroprotective effect of cyclosporine A, which effectively attenuates the excessive generation of reactive oxygen species and enhances the activation of mitogen-activated protein kinase and protein kinase C, is unequivocal in both in vitro and preclinical models (Pignataro et al., 2008; Xuwen et al., 2008). Additionally, cyclosporin A also hinders the activation of mPTP (Norbert et al., 2016). It has been observed that its inhibition on mPTP opening is achieved by binding to CypD (Davis et al., 2010). The compound has the ability to bind to CyPD, effectively inhibit its isomerase activity, and displace it from mPTP (Shohei et al., 2018). Cyclosporine A exhibits potent activity in inhibiting mPTP opening at low concentrations (0.2–1.2 μM). Inhibited mPTP can reduce tissue expression levels of cytochrome c and AIF while improving secondary damage factors such as brain edema, cortical apoptosis, and neurobehavioral defects in a rat model with ICH (Xie et al., 2012). The neuroprotective drug Edaravone, similar to cyclosporin A, is believed by some scholars to exert its protective mechanism through modulation of mPTP. This modulation inhibits upstream ROS events and subsequently prevents mPTP opening, ultimately leading to neuronal survival (Shohei et al., 2018).

Studies have revealed that mitochondria accumulate high concentrations of melatonin (Leon et al., 2004), and the substantial synthesis of melatonin in mitochondria plays a crucial role in maintaining cellular energy metabolism and mitochondrial function (He et al., 2016). Apart from its antioxidant effects, melatonin exerts neuroprotective actions by modulating mPTP activity and cell death processes (Petrosillo et al., 2009; Espino et al., 2010), which has emerged as a novel approach for intervening in mPTP-related secondary damage following ICH. Initial observations in rat brain astrocytes demonstrated that melatonin appeared to inhibit mitochondrial ROS formation and prevent cell death by targeting Ca^2+^-mediated mPT (Jou et al., 2010). Subsequent research found that when Ca^2+^ homeostasis was disrupted, melatonin effectively prevented mitochondrial depolarization and attenuated mPTP-induced neurotoxicity (Jou, 2011). Furthermore, the addition of melatonin to isolated brain mitochondria inhibited the opening of mPTP, suggesting its potential to impede mPTP-mediated mitochondrial dysfunction (Waseem et al., 2016). In animal models of ICH, melatonin mitigates mitochondrial dysfunction by upregulating antioxidants, thereby inhibiting the opening of mPTP to attenuate secondary damage following ICH and safeguard brain tissue (Wang Z. et al., 2018). Additionally, it has been observed that melatonin significantly diminishes mitochondrial swelling and membrane potential while enhancing mitochondrial respiration (Morciano et al., 2021). A recent mechanistic study demonstrated that antagonists of melatonin receptor 1 (MT1) nullify the inhibition of mPTP opening induced by melatonin, whereas MT1 agonists exert an opposite effect (Fang et al., 2020). However, the precise mechanism through which melatonin regulates mPTP remains unclear, with one possible molecular mechanism being the interaction between melatonin and CypD (Zhou et al., 2018).

The opening of mPTP will result in various forms of cell death, thereby inducing further mPTP opening and leading to more severe cellular damage. Abnormal mPTP opening can cause disruption in mitochondrial structure and function. Therefore, preserving the integrity of mitochondrial structure and function is crucial for preventing the progression of secondary damage following ICH. Adiponectin receptor 1 (AdipoR1) is widely expressed in neurons and its activation has a beneficial impact on damaged neurons (Song J. et al., 2015; Duan et al., 2016). Activation of AdipoR1 enhances AMP-activated protein kinase (AMPK) phosphorylation. As a downstream signaling molecule of AMPK, peroxisome proliferator-activated receptor-gamma coactivator-1 alpha (PGC1α) plays a pivotal role in regulating mitochondrial biosynthesis. In cases of ICH, PGC1α activation can stimulate the NRF1/TFAM axis (nuclear respiratory factor 1, NRF1; Mitochondrial transcription factor A, TFAM), thus improving mitochondrial DNA and ATP production (You et al., 2016). Furthermore, PGC1α activation also mitigates the collapse of mitochondrial membrane potential (Δψm) and reduces mitochondrial ROS production through a SIRT3-dependent mechanism (Zhang et al., 2016; Yu et al., 2017; Zheng et al., 2018). In conclusion, the Adipor1-AMPK-PGC1α axis may play a role in the progression of mitochondrial damage following ICH. Activation of AdipoR1 expression can mitigate secondary damage after ICH by preserving mitochondrial structure and function. SS31, an antioxidant peptide that targets mitochondria, has demonstrated beneficial effects in various diseases due to its potent antioxidant and neuroprotective properties. Studies have shown that SS31 ameliorates oxidative damage induced by ICH by reducing reactive oxygen species generation, alleviating lipid peroxidation, and enhancing antioxidant enzyme activity. Additionally, SS31 significantly attenuates neurological deficits, brain edema, neuronal apoptosis, and blood-brain barrier disruption after ICH. The specific mechanism involves activating the Nrf2 signaling pathway, increasing mtDNA copy number, and reversing mitochondrial dysfunction (Zhou et al., 2023). Previous studies have indicated that astaxanthin improves mitochondrial function in SH-SY5Y neuronal cells exposed to 1-methyl-4-phenylpyridine and suggests its potential as a therapeutic agent for neurodegenerative diseases (Lee et al., 2011). In an ICH rat model treated with astaxanthin in the cortex region, it was observed that Cyt C release from mitochondria was reduced along with down-regulation of Bax/Bcl-2 ratio and inhibition of caspase-3 activity. These changes in apoptosis-related indicators support the role of astaxanthin in improving mitochondrial function after ICH (Wang et al., 2019).

## 7 Summary

Mitochondria, being a crucial organelle in the body, actively participate in various cellular processes and are also exposed to diverse biochemical stimuli. Detrimental biochemical stimuli alter the cellular environment where mitochondria reside and induce a permeability transition in these organelles. With further research advancements, scholars have discovered that the transition of mitochondrial permeability is triggered by the opening of mPTP. The specific composition of mPTP remains controversial; however, several conformational models have been proposed by scholars including CypD, ANT, VDAC, PiC, ATP synthase, and SPG7 as potential candidates for mPTP composition. Studies have revealed that the opening of mPTP disrupts mitochondrial morphology and function while exacerbating cell damage. This mechanism may be associated with pathological processes such as oxidative stress, apoptosis, cell necrosis, autophagy, ferroptosis among others – all commonly observed after ICH. Therefore, targeting mPTP represents a promising treatment approach for improving secondary injury following ICH. However, current research primarily exists in animal experiments with limited clinical investigations conducted thus far. In future studies on both the composition of mPTP and its precise involvement in secondary injury after ICH are required to provide clearer directions for ICH treatment.

## Author contributions

JC: Writing−original draft. J-YL: Writing−review and editing. WZ: Investigation, Writing−review and editing.
