# Supplementary material for: Mechanism and treatment of intracerebral hemorrhage focus on mitochondrial permeability transition pore
Source: Front Mol Neurosci. 2024 Jul 31;17:1423132. doi: 10.3389/fnmol.2024.1423132 (PMC11328408; doi:10.3389/fnmol.2024.1423132)
Supplement: Supplementary file 1 [file Data_Sheet_1.docx]

Supplementary Material

**
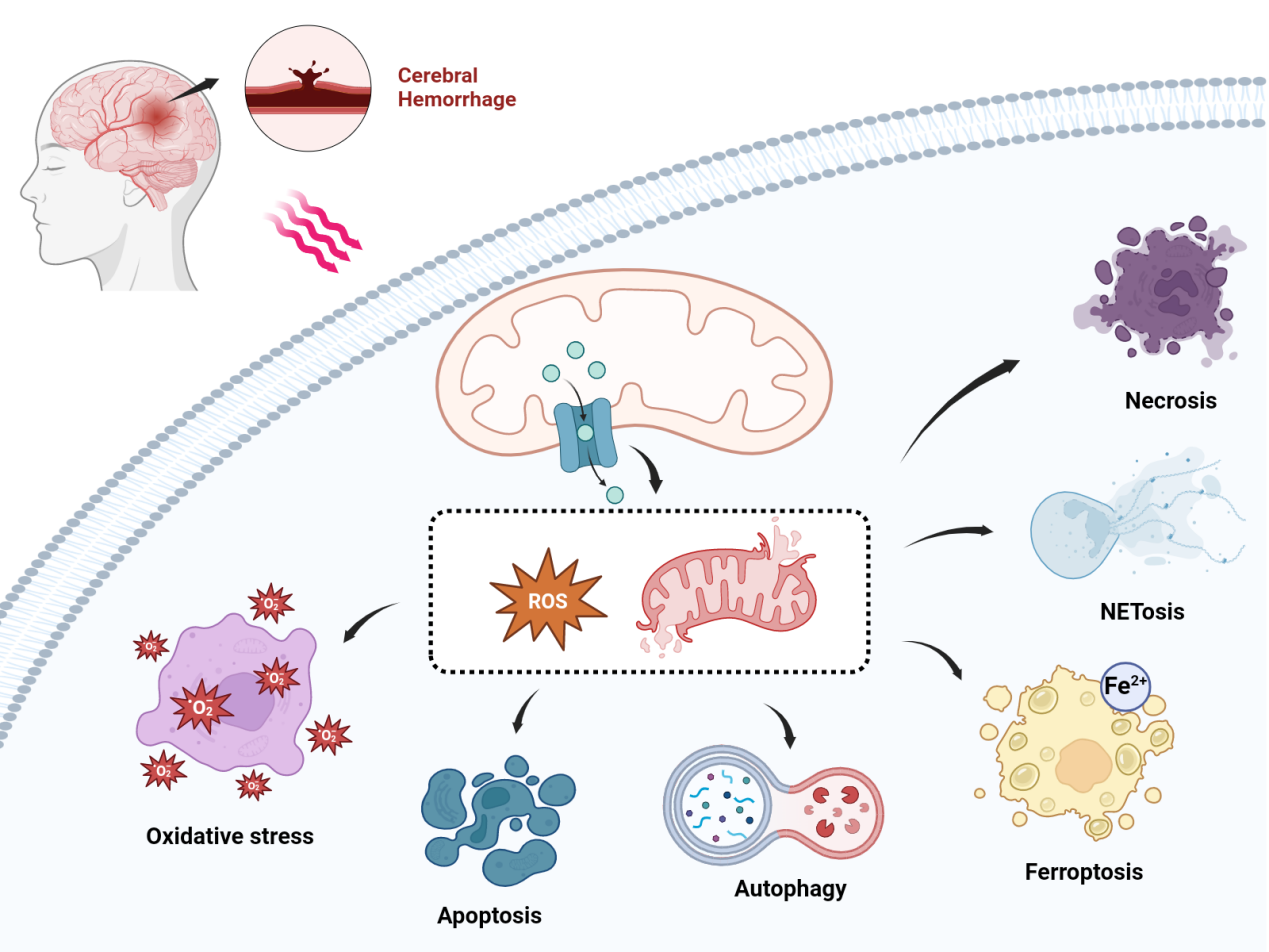
**

**Fig. 1 mPTP pathological process involved in secondary injury after ICH**

After ICH, the opening of mPTP occurs in an unfavorable cellular milieu, leading to aberrations in both mitochondrial morphology and function. This event triggers a cascade of pathological processes including oxidative stress, apoptosis, necrosis, autophagy, ferroptosis, and NETosis.

**References**

Bonora, M., A. Bononi, E. De Marchi, C. Giorgi, M. Lebiedzinska, S. Marchi, S. Patergnani, A. Rimessi, J. M. Suski, A. Wojtala, M. R. Wieckowski, G. Kroemer, L. Galluzzi and P. Pinton (2013). "Role of the c subunit of the FO ATP synthase in mitochondrial permeability transition." Cell Cycle **12**(4): 674-683.

Crompton, M., S. Virji and J. M. Ward (1998). "Cyclophilin-D binds strongly to complexes of the voltage-dependent anion channel and the adenine nucleotide translocase to form the permeability transition pore." Eur J Biochem **258**(2): 729-735.

Giorgio, V., S. von Stockum, M. Antoniel, A. Fabbro, F. Fogolari, M. Forte, G. D. Glick, V. Petronilli, M. Zoratti, I. Szabó, G. Lippe and P. Bernardi (2013). "Dimers of mitochondrial ATP synthase form the permeability transition pore." Proc Natl Acad Sci U S A **110**(15): 5887-5892.

Gutiérrez-Aguilar, M. and C. P. Baines (2015). "Structural mechanisms of cyclophilin D-dependent control of the mitochondrial permeability transition pore." Biochim Biophys Acta **1850**(10): 2041-2047.

Halestrap, A. P. and A. M. Davidson (1990). "Inhibition of Ca2(+)-induced large-amplitude swelling of liver and heart mitochondria by cyclosporin is probably caused by the inhibitor binding to mitochondrial-matrix peptidyl-prolyl cis-trans isomerase and preventing it interacting with the adenine nucleotide translocase." Biochem J **268**(1): 153-160.

He, L. and J. J. Lemasters (2002). "Regulated and unregulated mitochondrial permeability transition pores: a new paradigm of pore structure and function?" FEBS Lett **512**(1-3): 1-7.

Leung, A. W. and A. P. Halestrap (2008). "Recent progress in elucidating the molecular mechanism of the mitochondrial permeability transition pore." Biochim Biophys Acta **1777**(7-8): 946-952.

Leung, A. W., P. Varanyuwatana and A. P. Halestrap (2008). "The mitochondrial phosphate carrier interacts with cyclophilin D and may play a key role in the permeability transition." J Biol Chem **283**(39): 26312-26323.

Mnatsakanyan, N., M. C. Llaguno, Y. Yang, Y. Yan, J. Weber, F. J. Sigworth and E. A. Jonas (2019). "A mitochondrial megachannel resides in monomeric F(1)F(O) ATP synthase." Nat Commun **10**(1): 5823.

Shanmughapriya, S., S. Rajan, N. E. Hoffman, A. M. Higgins, D. Tomar, N. Nemani, K. J. Hines, D. J. Smith, A. Eguchi, S. Vallem, F. Shaikh, M. Cheung, N. J. Leonard, R. S. Stolakis, M. P. Wolfers, J. Ibetti, J. K. Chuprun, N. R. Jog, S. R. Houser, W. J. Koch, J. W. Elrod and M. Madesh (2015). "SPG7 Is an Essential and Conserved Component of the Mitochondrial Permeability Transition Pore." Mol Cell **60**(1): 47-62.

Urbani, A., V. Giorgio, A. Carrer, C. Franchin, G. Arrigoni, C. Jiko, K. Abe, S. Maeda, K. Shinzawa-Itoh, J. F. M. Bogers, D. G. G. McMillan, C. Gerle, I. Szabò and P. Bernardi (2019). "Purified F-ATP synthase forms a Ca(2+)-dependent high-conductance channel matching the mitochondrial permeability transition pore." Nat Commun **10**(1): 4341.
